# Development of a quantitative NS1-capture enzyme-linked immunosorbent assay for early detection of yellow fever virus infection

**DOI:** 10.1038/s41598-017-16231-6

**Published:** 2017-11-24

**Authors:** Taissa Ricciardi-Jorge, Juliano Bordignon, Andrea Koishi, Camila Zanluca, Ana Luiza Mosimann, Claudia Nunes Duarte dos Santos

**Affiliations:** Laboratório de Virologia Molecular, Instituto Carlos Chagas, FIOCRUZ-PR, Curitiba, Paraná Brazil

## Abstract

Yellow fever is an arboviral disease that causes thousands of deaths every year in Africa and the Americas. However, few commercial diagnostic kits are available. Non-structural protein 1 (NS1) is an early marker of several flavivirus infections and is widely used to diagnose dengue virus (DENV) infection. Nonetheless, little is known about the dynamics of Yellow fever virus (YFV) NS1 expression and secretion, to encourage its use in diagnosis. To tackle this issue, we developed a quantitative NS1-capture ELISA specific for YFV using a monoclonal antibody and recombinant NS1 protein. This test was used to quantify NS1 in mosquito and human cell line cultures infected with vaccine and wild YFV strains. Our results showed that NS1 was detectable in the culture supernatants of both cell lines; however, a higher concentration was maintained as cell-associated rather than secreted into the extracellular milieu. A panel of 73 human samples was used to demonstrate the suitability of YFV NS1 as a diagnostic tool, resulting in 80% sensitivity, 100% specificity, a 100% positive predictive value and a 95.5% negative predictive value compared with RT-PCR. Overall, the developed NS1-capture ELISA showed potential as a promising assay for the detection of early YF infection.

## Introduction

Yellow fever (YF) is an arboviral disease transmitted by mosquitoes of the genera *Aedes* sp., *Haemagogus* sp. and *Sabethes* sp. and is endemic in many African and South American countries^1^. Despite the existence of an effective vaccine, thousands of cases of YF are reported every year. A recent study estimates the annual occurrence of 51,000–380,000 severe cases of yellow fever and 19,000–180,000 deaths due to this disease in Africa alone^2^. Since December 2016, several Brazilian states have experienced an important epizootic YF outbreak, with almost eight hundred confirmed human cases and more than two hundred deaths as of May 2017^3^.

Yellow fever virus (YFV) belongs to the *Flavivirus* genus within the *Flaviviridae* family. YFV is an enveloped virus with a positive-sense RNA genome that encodes three structural proteins (C, M, and E) and seven non-structural proteins (NS1, NS2a, NS2b, NS3, NS4a, NS4b, and NS5) in a single open reading frame^4^. The *Flavivirus* non-structural protein 1 (NS1) is a glycoprotein approximately 48 kDa in size that is found in the inner and outer membranes of infected cells^5,6^. NS1 is also secreted into the extracellular space (sNS1) as a barrel-shaped homo-hexamer associated with lipids; the formation and secretion of this structure are strongly dependent on the amino acid sequence and glycosylation state of NS1^7–11^. The presence of sNS1 in the sera of patients infected with West Nile virus (WNV) and dengue virus (DENV) is used as an early marker of infection^12–14^. However, in contrast to other flaviviruses, little is known about the dynamics of the expression and secretion of YFV NS1^7,15,16^. Therefore, the potential of NS1 as an early marker of infection remains unknown.

To address this issue, the present study aimed to develop a specific NS1-capture ELISA to investigate the production and secretion patterns of YFV NS1 in vertebrate and mosquito cell lines and to evaluate its applicability as an early diagnostic test for acute YFV infection in humans. A panel of sera from patients naturally infected with YFV was used as a proof-of-concept.

## Results

### Recombinant NS1 protein and monoclonal antibody characterization

**Figure 1:**
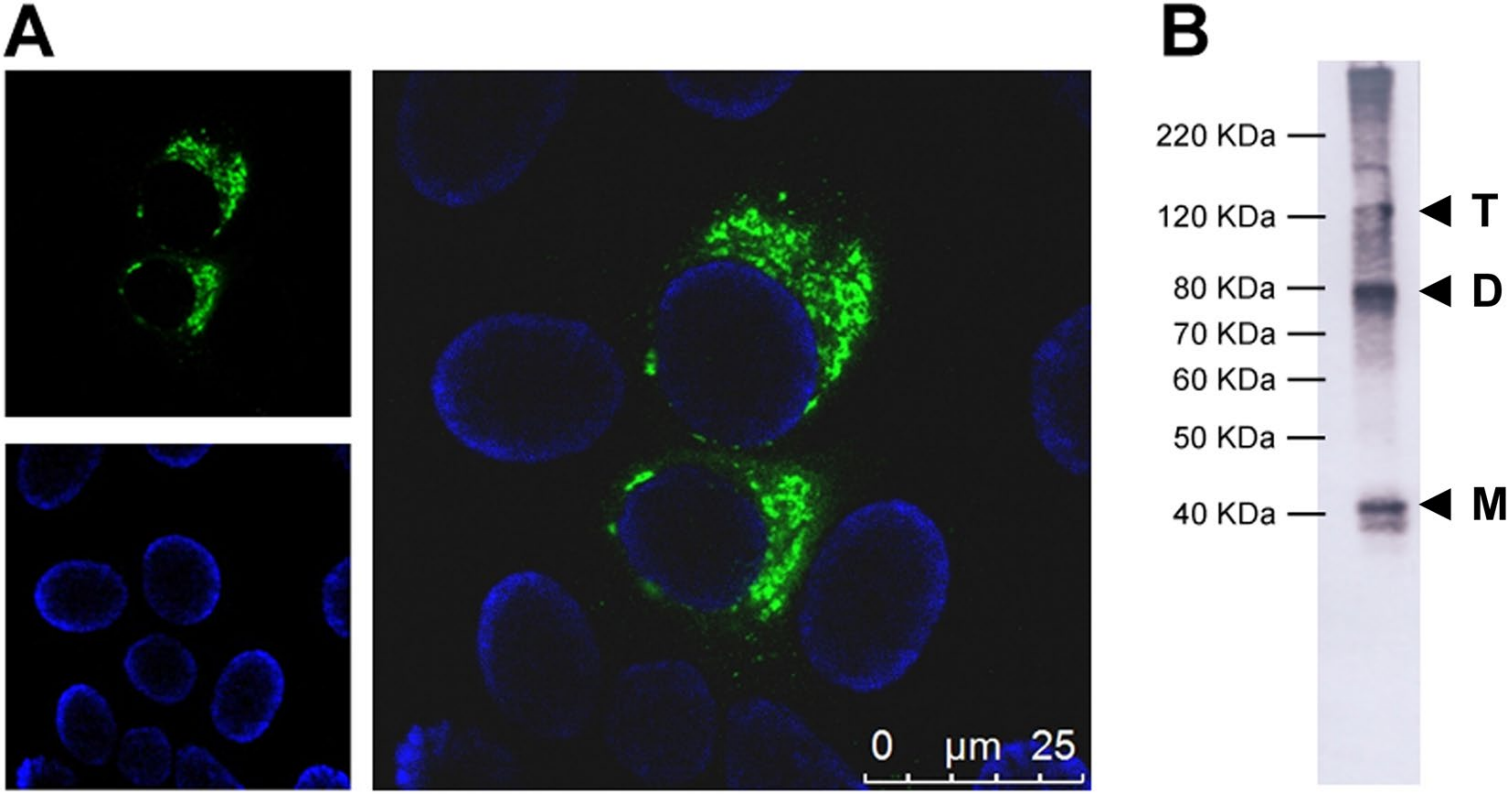
Characterization of 3A8-C12 MAb reactivity. (**A**) Indirect immunofluorescence of YFV/17DD-infected Huh7.5 cells (confocal microscopy) stained with the 3A8-C12 monoclonal antibody plus anti-mouse IgG conjugated with AlexaFluor 488. DNA was stained using DAPI; (**B**) Western blotting analysis of rNS1 resolved by 15% SDS-PAGE. The membrane was stained with the 3A8-C12 monoclonal antibody, followed by anti-mouse IgG conjugated with alkaline phosphatase. Solid arrows indicate oligomeric forms of YFV rNS1: (M) monomers, (D) dimers and (T) trimers.

The generation of the 3A8-C12 YFV NS1-specific monoclonal antibody was previously described^17^. The isotyping test revealed that the antibody was an IgG1-κ isotype. Indirect immunofluorescence of YFV/17DD-infected Huh7.5 cells showed granular staining at the perinuclear location typical of the NS1 protein (Fig. 1a). Western blotting analysis of rNS1 indicated that 3A8-C12 recognized a linear epitope (Fig. 1b).

**Figure 2:**
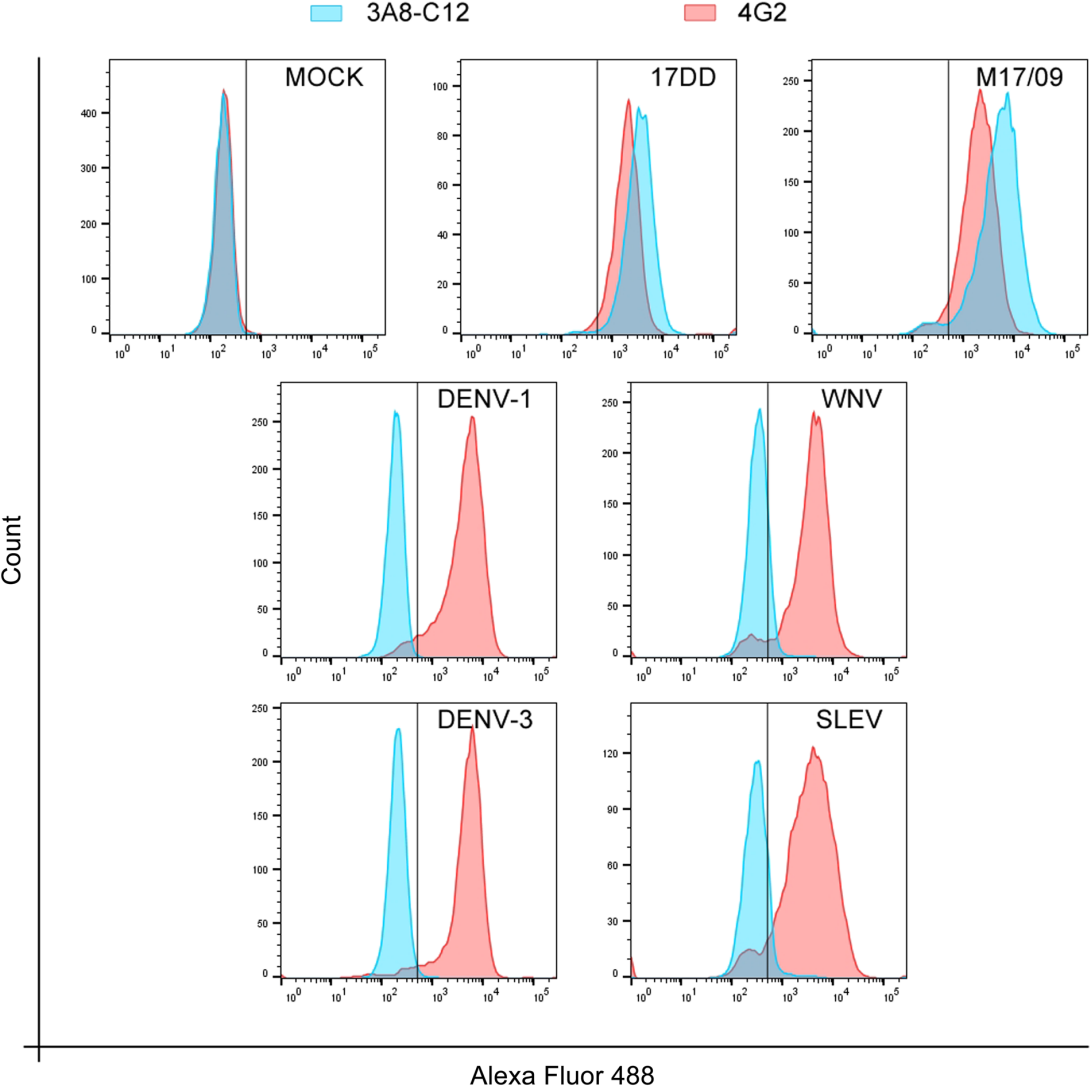
Flow cytometry analysis of the cross-reactivity of the 3A8-C12 MAb against different flaviviruses. Huh7.5 cells were infected with YFV, DENV, SLEV, and WNV and then immunostained. The 4G2 MAb against the *Flavivirus* E protein was used as a positive control, and uninfected cells (MOCK) were used as the negative control. The plotted data are representative of two independent experiments. The cell suspensions were analyzed by flow cytometry on a FACSCanto II (BD Biosciences, San Jose, California, USA). All flow cytometry assay data were analyzed in FlowJo v7.1 (TreeStar Inc., Ashland, Oregon, USA).

To ensure the specificity of the selected monoclonal antibody, a flow cytometry analysis was performed with cells infected with other flaviviruses (DENV, SLEV, and WNV). In this test, the 3A8-C12 MAb exhibited no cross-reactivity with the tested viruses and was able to detect the vaccine and wild strains of YFV with a similar sensitivity as the positive control for flavivirus infection (4G2) (Fig. 2). Likewise, no cross-reaction was detected when ZIKV infected cells were tested (data not shown).

### Development of a YFV NS1-capture ELISA

**Figure 3:**
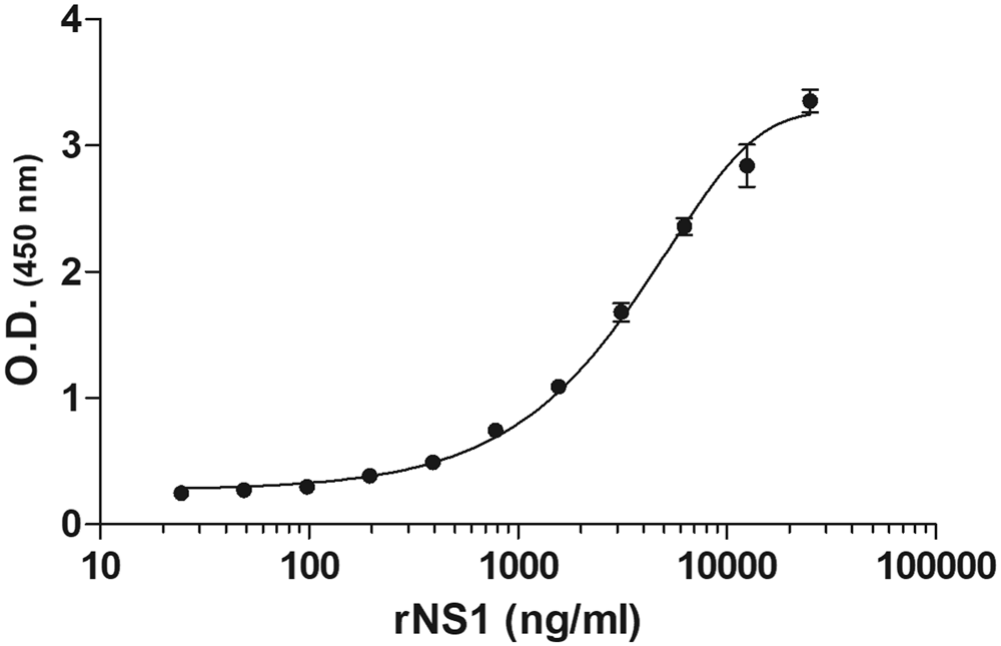
YFV NS1-capture ELISA. The NS1-capture ELISA standard curve with different concentrations of the recombinant YFV NS1 protein (rNS1). The assay values represent three experiments, and the error bars indicate standard deviation.

The 3A8-C12 monoclonal antibody was used as a capture and detection antibody, and rNS1 produced in insect cells was used as the internal standard to develop a YFV NS1-capture ELISA. Serial dilutions of purified rNS1 from 100 μg/ml to 6.1 ng/ml were used to determine the assay sensitivity. Values for the protein concentration and optical density at 450 nm were plotted for the non-linear regression calculations. The lowest concentration at which rNS1 can be reliably quantified with this test is 24.4 ng/ml, and a concentration-dependent response is observed up to 12.5 μg/ml (Fig. 3). This range was used as a standard curve for interpolation of the sample values.

### Kinetics of NS1 production and secretion by human and mosquito cell lines

**Figure 4:**
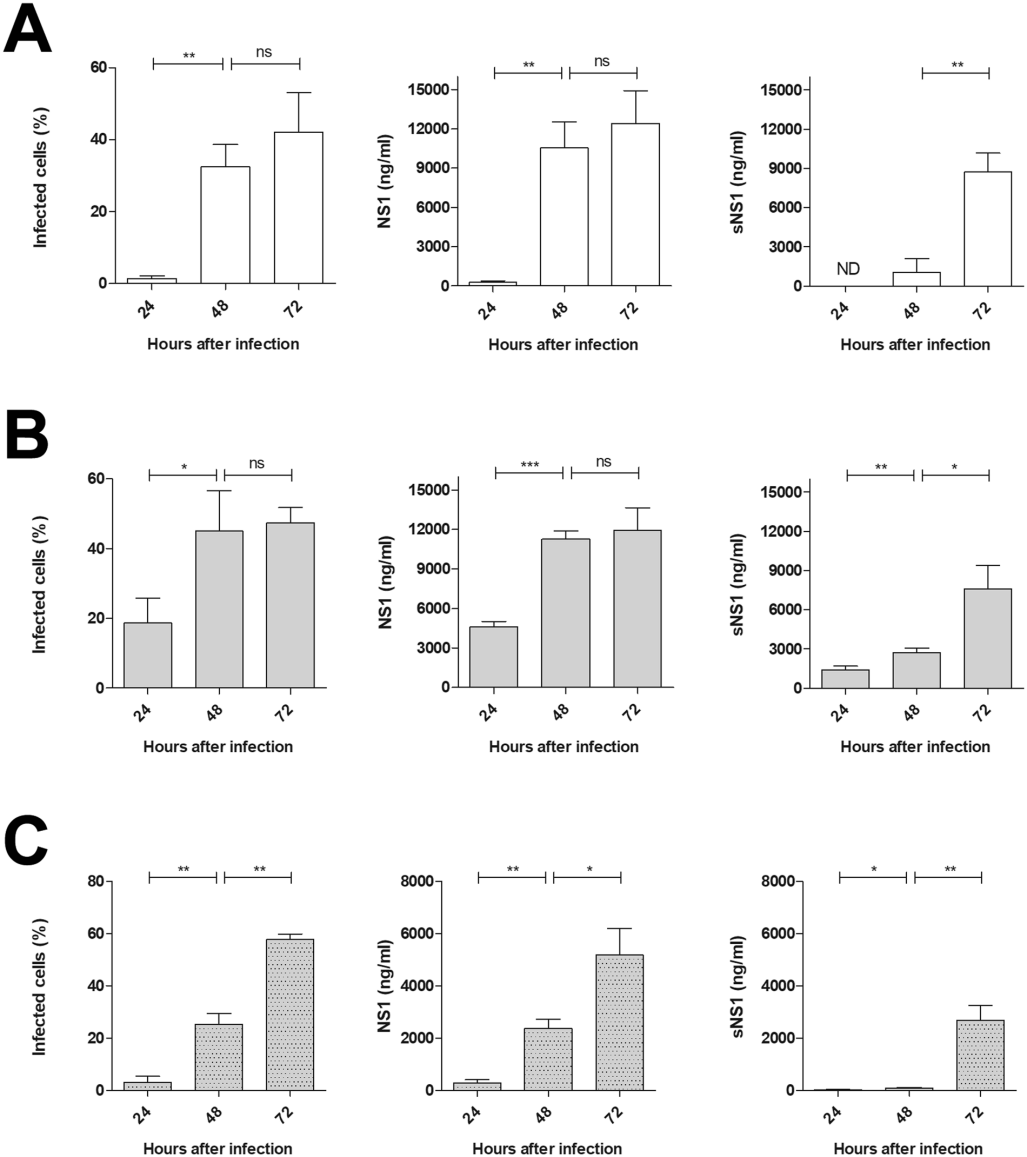
Kinetics of cell infection and NS1 production and secretion. Percentage of infected cells (left panel) and dosage of NS1 in cell lysates (middle panel) and culture supernatant (right panel) from (**A**) Huh7.5 cells infected with YFV vaccine strain (17DD); (**B**) Huh7.5 cells infected with YFV wild strain (M17/09); and (**C**) C6/36 cells infected with YFV wild strain (M17/09), all at MOI of 0.125. All sNS1 measurements under the limit of sensibility were considered negative (ND). Statistical analysis performed with unpaired t-test with Welch’s correction: ns if p > 0.05; * if p ≤ 0.05; ** if p ≤ 0.01; *** if p ≤ 0.001. Data represent the mean ± SD of three technical replicates.

Huh7.5 and C6/36 cells infected with YFV were used to study NS1 synthesis and secretion over time in a three-point kinetic assay. At 24, 48 and 72 hours after infection, the culture supernatant and cell lysate were analyzed using the developed YFV NS1-capture ELISA. Parallel cell cultures were used to determine the percentage of infected cells by flow cytometry at the same time points (Fig. 4).

Huh7.5 cells infected with the vaccine and wild YFV strains (17DD and M17/09, respectively) showed comparable levels of synthesized and secreted NS1 protein at 48 and 72hdpi hours after infection. The Huh7.5 cell cultures could not be evaluated beyond 72 hours post-infection, because both strains caused high cytopathic effects in this cell line, leading to cell lysis. Nevertheless, the level of secreted NS1 increased over time, whereas the level of cell-associated NS1 hit a plateau, probably due to cell death. Because the vaccine strain could not properly infect insect cells, the C6/36 cells were infected only with the wild strain. In these cells, NS1 synthesis and secretion were also proportional to viral infection, but they occurred at a lower rate than in the mammalian cells.

### Viability of the YFV/17DD-infected cell lines

**Figure 5:**
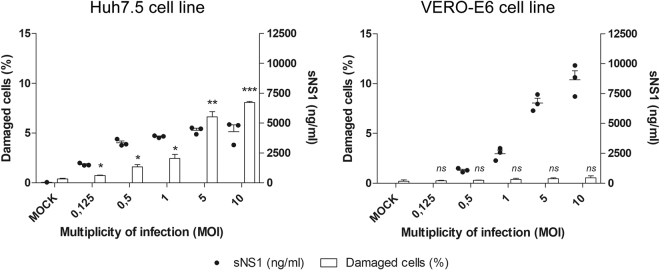
Cell viability and soluble NS1 secretion. Dosage of NS1 in culture supernatant and determination of cell membrane lysis of Huh7.5 cells or VERO-E6 infected with YFV vaccine strain (17DD) with different MOIs at 48 hour after infection. Percentages of damaged cells was determined with 7-AAD reagent. Statistical analysis performed against mock values using unpaired t-test with Welch’s correction: ns if p > 0.05; * if p ≤ 0.05; ** if p ≤ 0.01; *** if p ≤ 0.001. Statistical analysis does not include sNS1 measurements since mock samples were under the limit of sensibility, hence considered negatives. Data represent the mean ± SD of three technical replicates.

Because YFV is highly cytopathic, especially in mammalian cells such as Huh7.5, the NS1 detected in the cell culture supernatant could have originated from cell lysis and not from active secretion. To investigate this hypothesis, Huh7.5 and VERO-E6 cells were infected with variable MOIs of YFV/17DD and analyzed 48 hours post-infection. Soluble NS1 was measured in the culture supernatant, whereas the cells were analyzed with the Operetta® High Content Imaging System to determine the percentage of cells with damaged membranes stained with 7-AAD (Fig. 5).

Overall, the results refuted the hypothesis of the detection of NS1 due to cell lysis and showed higher levels of soluble NS1 in the VERO-E6 cell culture. Notably, YFV causes only a discrete cytopathic effect in VERO-E6 cells at 48 hours post-infection compared to Huh7.5 cells. These results indicate that the soluble NS1 may originate from cell secretion rather than overflow of cellular content after cell lysis caused by viral replication. Indeed, higher cell viability might allow viral replication for longer periods and at higher levels, leading to the accumulation of secreted NS1 in the cell culture supernatant.

### Testing of human samples

A panel of 73 human samples provided by the Evandro Chagas Institute (IEC, Brazil) was used to evaluate the suitability of the developed NS1-capture ELISA for the diagnosis of YFV acute phase infection (Table 1). The YFV NS1-capture ELISA was able to detect 12 out of the 15 YFV RT-PCR-positive samples. No cross-reactivity with other related arboviruses (DENV, ZIKV and CHIKV) was observed. Likewise, no false positive results were observed when testing the control negative sera. Since NS1 is an early biomarker of flavivirus infection that is usually positive simultaneously with the viremia^17^, only RT-PCR-positive samples were considered for the assessment of the test performance. Thus, the YFV NS1-capture ELISA presented 80% sensitivity, 100% specificity, a 100% positive predictive value (PPV), and a 95% negative predictive value (NPV). The final concentration of sNS1 in the positive samples varied from 177.6 to 4597.6 ng/ml (Table 2). The negative samples were below the limit of detection (24.4 ng/ml).

**Table 1:** YFV-NS1 detection in human serum samples.

**Group**	NS1-capture ELISA		
	***n***	**Positive**	**Negative**
YFV RT-PCR positive	15	12	3
DENV RT-PCR positive	14	0	14
ZIKV RT-PCR positive	14	0	14
CHIKV RT-PCR positive	15	0	15
Negative sera	15	0	15

n: number of samples.

**Table 2:** NS1 quantification in serum samples from patients with YFV viremia.

Serum sample	Days after symptom onset	YFV NS1 (ng/ml)
1	2	4597.6
2	0	4029.9
3	2	3949.8
4	0	762.0
5	6	177.6
6	9	284.5
7	3	1280.2
8	4	—
9	7	816.4
10	5	4071.7
11	7	629.0
12	7	—
13	3	908.3
14	4	—
15	9	515.3

:negative.

## Discussion

The Flavivirus NS1 protein is found cell-associated to inner and outer membranes of infected cells and secreted into the extracellular space (sNS1) as a barrel-shaped homo-hexamer associated with lipids^6,7,10,11^. After secretion, NS1 can also be endocytosed by and attached to the surface of non-infected cells^18,19^. Intracellular NS1 has been shown to take part in the RNA replication process^20,21^. On the other hand, YFV sNS1 may play important roles in immune evasion mechanisms through interactions with the complement component C4 and C4b binding protein^22,23^. Nonetheless, its role in yellow fever infection has yet to be further studied. NS1 has been successfully used as an early marker of DENV infection since its discovery^12^. Sensitive NS1-capture ELISAs have been developed to diagnose WNV and JEV infections^13,14,24^, but their use for the diagnosis of clinical samples remains underutilized or unreported. In contrast, little is known about the dynamics of YFV NS1 synthesis and secretion. An early study showed the presence of soluble NS1 in culture supernatants of YFV-infected mammalian cell lines and the existence of different glycosylation patterns between cell-associated and soluble NS1^7^. Another study reported the lack of YFV NS1 secretion by insect cells^16^. However, the potential use of YFV NS1 detection for diagnostic purposes appeared in the literature only in a recent study, although the secreted protein was not quantified^25^.

In the present study, we developed a YFV NS1-capture ELISA to investigate the pattern of protein secretion by YFV-infected cells and to explore the possibility of its use for clinical diagnosis. This test uses a specific monoclonal antibody for capture and detection to favor the detection of oligomeric forms of the NS1 protein. The YFV NS1-capture ELISA has a limit of detection and quantification of 24.4 ng/ml of protein. The achieved sensitivity is comparable with commercial and in-development tests for other flaviviruse*s* ^12,13,24,26,27^ and is sufficient to quantify the accumulation of the NS1 protein in cell culture supernatants. The total amount of detectable YFV sNS1 was comparable to the amounts found for WNV under similar conditions^14^. Our study also demonstrated that a higher NS1 concentration was maintained as cell-associated rather than secreted into the extracellular milieu.

Previous studies have reported the lack of NS1 secretion by insect cells^16,28^. This finding was hypothesized to be due to a different protein glycosylation pattern than the NS1 detected in infected mammalian cells^6,8,28^. However, these studies have analyzed cell culture supernatants using less sensitive methods and at very early time points after infection. Our results showed that C6/36 cells were able to secrete YFV NS1 when infected with the wild M17/09 strain, albeit in lower amounts compared to vertebrate cells; additionally, the secreted NS1 was detectable only when analyzed at later time points (after 48 hours post-infection). In agreement, previous studies reported DENV NS1 secretion by insect cell lines^25,26^.

The soluble YFV NS1 detected by the assay presented here is suggestive to have originated from active cell secretion, and its levels seem to depend on the cell line and multiplicity of infection. Our results show an increased amount of soluble NS1 in the culture supernatant from the Huh7.5 cell line at lower MOIs (0.125 and 0.5, which would resemble natural infection). At higher MOIs, a marked cytopathic effect was observed, and the NS1 protein detected in the cell supernatant could also be due to cell lysis. VERO–E6 cells are less susceptible to viral cytopathic effects; thus, cell integrity might allow viral replication to precede for longer times and at higher levels, leading to the accumulation of secreted NS1 in the cell culture supernatant.

Finally, tests with human samples were conducted to investigate the applicability of sNS1 detection for the diagnosis of YFV infection. The preliminary experiments failed to detect NS1 in the sera or plasma of immunized individuals from 3 to 10 days after vaccination with the Brazilian 17DD vaccine (data not shown). However, in tests with a panel of naturally infected human samples, the YFV NS1-capture ELISA presented a promising performance in terms of the specificity, NPV and PPV. The sensitivity of the assay was 80%, which was sufficient as a proof-of-concept regarding its utility in diagnosis. The settings of the assay may still be improved through further tests with a larger panel comprising well characterized samples in terms of viremia, clinical manifestations and immune status.

In conclusion, the developed NS1-capture ELISA showed potential as a promising tool for the early detection of YFV infection and could be a useful reagent for diagnostic surveillance during yellow fever outbreaks, such as the outbreak that occurred recently in Angola (2015–2016) and the ongoing outbreak in Brazil (2016–2017), especially under the current scenario of the circulation of multiple arboviruses.

## Materials and Methods

### Cells and Viruses

Huh7.5 (ATCC® PTA-8561TM; Manassas, Virginia, USA) and VERO-E6 (Sigma, 85020206; Salisbury, UK) cells were cultivated in Dulbecco’s modified Eagle’s medium/nutrient F-12 Ham (Gibco, Grand Island, NY, USA) with 7% fetal bovine serum (FBS; Gibco, Grand Island, NY, USA), 100 U/ml of penicillin, and 100 μg/ml of streptomycin (P/S) (Gibco, Grand Island, NY, USA). C6/36 *Aedes albopictus* cells (ATCC CRL-1660; Manassas, Virginia, USA) were cultured in Leibovitz’s L15 medium (Gibco, Grand Island, NY, USA) with 5% FBS, 0.26% tryptose (Gibco, Grand Island, NY, USA), and 25 μg/ml of gentamicin (Gibco, Grand Island, NY, USA). Recombinant S2 cells (Thermo Fisher Scientific, R69007) were cultivated in Schneider’s Drosophila Medium (Gibco, Grand Island, NY, USA) with 7% FBS and 100 U/ml and 100 µg/ml of P/S and induced by the addition of 700 μM CuSO_4_. The YFV-17DD vaccine strain stock was obtained from two passages of the commercial vaccine (Biomanguinhos, Rio de Janeiro, Brazil) in Huh7.5 cells. The M17/09 YF wild strain^29^ corresponds to the second passage in C6/36 cells after virus isolation. Saint Louis encephalitis virus (SLEV) and WNV were propagated in VERO-E6 cells, whereas DENV-1 and DENV-3 were propagated in C6/36 cells. The multiplicity of infection (MOI) was determined from titers obtained in the same cell line used for the infection assays except for the VERO-E6 cells, for which titers from Huh7.5 cells were used. All assays which involved the infection of cells with either SLEV, WNV or the M17/09 YF wild strain were carried out under BSL-3 containment. The remaining experiments were carried under BSL-2 or BSL-1 containment in accordance with biosafety guidelines.

### Recombinant NS1 production

The NS1 gene sequence based on the 17DD strain (GenBank accession number U17066.1) was optimized for expression in *Drosophila melanogaster* cells (GenScript, Piscataway, NJ, USA) and cloned into pMT/BiP/V5-His A (Drosophila Expression kit, ThermoFisher, Waltham, Massachusetts, USA) to be expressed in trimeric form. S2 cells were transfected using CaCl_2_ and selected with 15 μg/ml of blasticidin for 2 weeks. Recombinant NS1 (rNS1) was produced after induction of the cells with 700 µM CuSO_4_ for 48 h. The harvested cells were lysed with 17 µl of cell lysis buffer (50 mM Tris, pH 7.8, 150 mM NaCl, and 1% NP-40) per cm^2^ of grown culture. The rNS1 was purified from the culture supernatant and cell lysate using Ni-NTA Agarose (Qiagen, Valencia, California, USA). The protein concentration was measured with the fluorometric method (Qubit®, Life Technologies, Carlsbad, California, USA).

### Monoclonal antibody production, purification and conjugation

The procedure used to obtain monoclonal antibody (MAb) 3A8-C12 directed against YFV NS1 was previously described^17^. Once screened and selected, the MAb was precipitated from hybridoma culture supernatant with a saturated ammonium sulfate solution and purified by affinity chromatography with a HiTrap Protein G HP column (GE Healthcare, Little Chalfont Bucks, UK). The MAb was conjugated with horseradish peroxidase (HRP) following a previously described protocol with minor modifications^30^. Briefly, instead of re-purification in a G protein column after conjugation, the MAb was precipitated with a saturated ammonium sulfate solution, resuspended and dialyzed in PBS, and diluted 1:1 in Guardian Peroxidase Conjugate Stabilizer/Diluent (Thermo Fisher Scientific, Waltham, Massachusetts, USA) for storage.

### NS1-capture ELISA

ELISA modules (Nalge Nunc International, Rochester, NY, USA) were coated with 500 ng of purified 3A8-C12 monoclonal antibody in carbonate buffer (0.05 M. pH 9.6) and incubated overnight at 4 °C. Non-specific binding was blocked by incubating with 300 μl/well of blocking buffer (PBS with 10% fetal bovine serum) for 1 h at 37 °C. Then, 100 μl/well of each sample was incubated for 1 hour at 37 °C, followed by 100 μl/well of 3A8-C12 conjugated with HRP diluted 1:800 in PBS. Each step was followed by rinsing 3x with washing buffer (PBS with 0.01% Tween 20). The reaction development started after the addition of 50 μl/well of TMB (KPL) for 15 min and stopped with the addition of 50 μl/well of 2 M H_2_SO_4_. The net value of the optical density at 450 nm was set after subtraction of the negative control value. The sNS1 concentration in the samples was calculated through interpolation from a standard curve built using serial dilutions of purified rNS1 (100 μg/ml −6.1 ng/ml).

### Kinetics of NS1 expression and secretion

Twenty-four-well plates containing 2 × 10^5^ Huh7.5 or C6/36 cells per well were infected with the YFV/17DD or YFV/M17-09 strain at an MOI of 0.125 for 1.5 hour. After virus adsorption, these inocula were replaced with 600 μl of culture medium, and the cells were maintained at 37 °C/5% CO_2_ (Huh7.5) or 28 °C (C6/36). Three wells were harvested at each time point (24, 48 and 72 hours after infection). NS1 was quantified in the cell culture supernatant and cell lysate using the NS1-capture ELISA. Culture supernatants were directly analyzed, whereas the harvested cells were lysed with 600 µl of cell lysis buffer prior to analysis. Statistical analysis were performed with GraphPad Prism version 5.00 for Windows (GraphPad Software, San Diego California USA) using unpaired t-test and Welch’s correction, assuming a confidence interval of 95%.

### Flow cytometry quantification

For viral protein labeling through flow cytometry, cells were harvested, washed with PBS, centrifuged then resuspended in 100 µL Cytofix/Cytoperm (BD Biosciences, San Jose, California, USA) and incubated for 20 min at room temperature, protected from light. After incubation, the cells were washed with 100 µL of Perm/Wash Solution 1x (BD Biosciences, San Jose, California, USA), centrifuged, and the supernatant was discarded. The cells were mixed with 100 µL of the anti-YFV NS1 MAb (3A8-C12) or the anti-Flavivirus Mab (4G2) diluted in Perm/Wash and incubated for 30 min at 37 °C. After incubation, the cells were washed with 100 µL Perm/Wash solution, mixed with 100 µL of AlexaFluor 488-conjugated goat anti-mouse IgG (Invitrogen, Carlsbad, California, USA) at 1:400 v/v in Perm/Wash, and incubated for 30 min at 37 °C. Then, the cells were washed twice with PBS and resuspended in PBS for the FACS analysis.

### Cell viability assay

Twenty-four-well plates containing 2 × 10^5^ Huh7.5 or VERO-E6 cells per well were infected with the YFV/17DD strain at various multiplicities of infection (three wells per MOI) for 1.5 hours. After virus adsorption, these inocula were replaced with 600 μl of culture medium, and the cells were maintained at 37 °C/5% CO_2_ for 48 hours. The culture supernatants were analyzed by YFV NS1-capture ELISA. The cells were washed in PBS, co-stained with 7-AAD (BD) and Vybrant® DyeCycle™ Violet Stain (Thermo Fisher Scientific, Waltham, Massachusetts, USA) and then analyzed with the Operetta® High Content Imaging System and Harmony Software (PerkinElmer, Waltham, Massachusetts, USA). Briefly, the total cell number per image was determined based on the Vibrant Violet-stained nuclei, and the number of dead cells or cells with membrane damage was determined based on the cells with 7-AAD-stained nuclei. Statistical analysis were performed with GraphPad Prism version 5.00 for Windows (GraphPad Software, San Diego California USA) using unpaired t-test and Welch’s correction, assuming a confidence interval of 95%.

### Testing of human samples

A panel of YFV-positive and -negative human serum samples kindly provided by the Evandro Chagas Institute (IEC, Brazil) was used as a proof-of-concept for the NS1-capture ELISA. The panel comprised 15 serum samples from patients with yellow fever, 14 serum samples from patients with dengue, 14 serum samples from patients with ZIKV infection, 15 serum samples from patients with Chikungunya virus (CHIKV) infection and 15 negative control serum samples. The positive samples were diagnosed by RT-PCR. To perform the test, 100 μl/well of sera diluted 1:50 (in PBS with 0.05% Tween 20 and 10% FBS) was used. The subsequent procedures followed the NS1-capture ELISA protocol described above. All samples were tested in triplicate. All experimental protocols were approved by Fiocruz and the Brazilian National Ethic Committee of Human Experimentation under the number CAAE: 10336512.4.0000.5248.

Authors confirm that all experiments were performed in accordance with relevant guidelines and regulations and when pertinent informed consent was obtained from all subjects.

## Electronic supplementary material

Supplementary Information
