# Supplementary material for: Development of a quantitative NS1-capture enzyme-linked immunosorbent assay for early detection of yellow fever virus infection
Source: Sci Rep. 2017 Nov 24;7:16229. doi: 10.1038/s41598-017-16231-6 (PMC5701136; doi:10.1038/s41598-017-16231-6)
Supplement: Supplementary file 1 — Supplementary Information [file 41598_2017_16231_MOESM1_ESM.pdf]

**Development of a quantitative NS1-capture enzyme-linked immunosorbent assay for early detection of yellow fever virus infection**

Taissa Ricciardi-Jorge, Juliano Bordignon, Andrea Cristine Koishi, Camila Zanluca, Ana Luiza Pamplona Mosimann and Claudia Nunes Duarte dos Santos*.

Laboratório de Virologia Molecular, Instituto Carlos Chagas, FIOCRUZ-PR, Curitiba, Paraná, Brazil

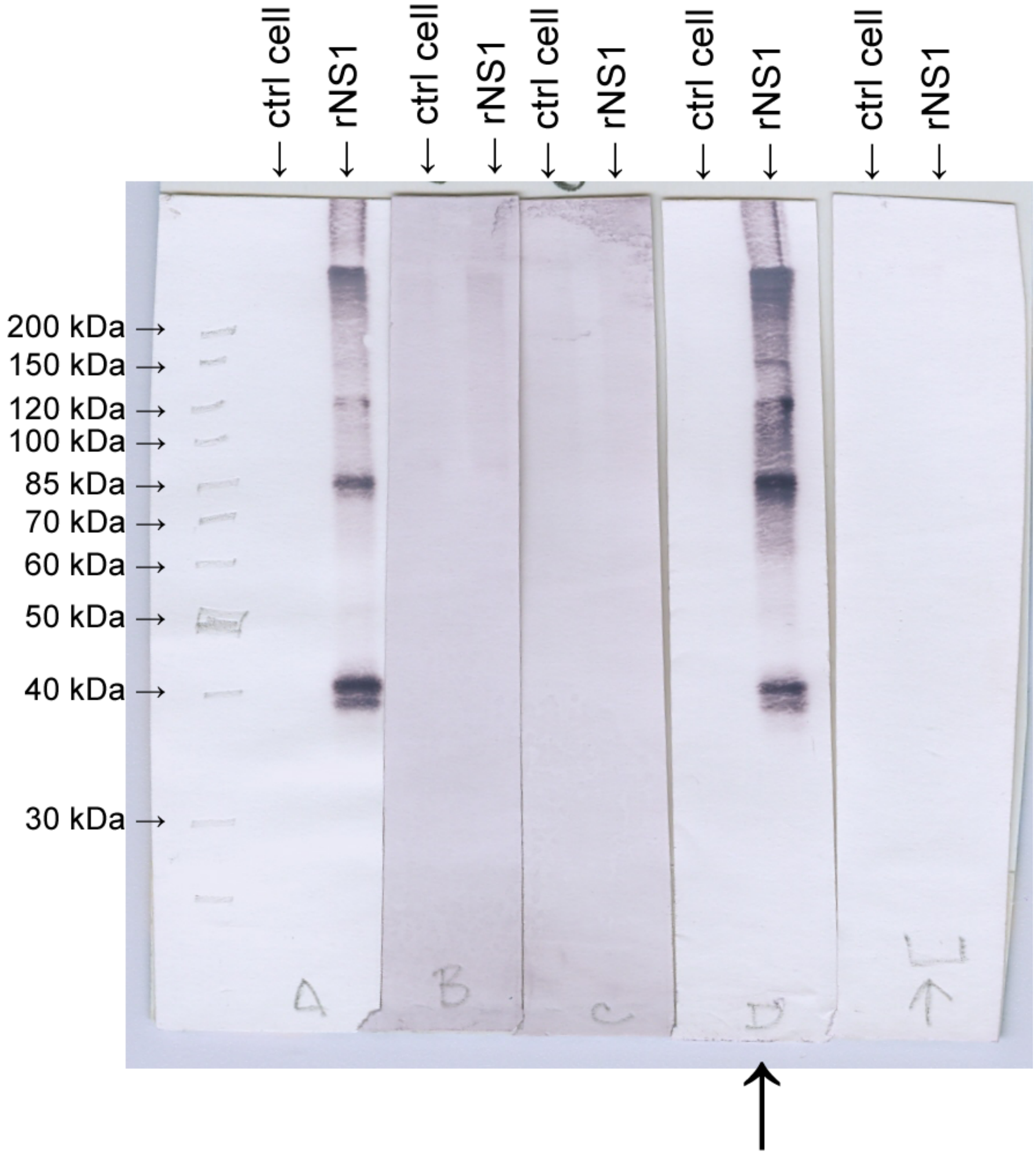

**Fig. S1- Original image of western-blot depicted in Figure 1**: Western-blot analysis for antibody reactivity testing. Control S2 cell (non transfected) and S2 cell transfected with pMT/BiP/V5-His A vector cloned with gene for expression of YFV NS1 protein (rNS1); samples were lysate in cell lysis buffer, resolved in SDS-PAGE and blotted. Each pair of samples were cut in strips for reaction with different non-related antibodies. Strip A: anti-V5 tag monoclonal antibody (Invitrogen); strips B, C and E: other antibodies (isotype controls); strip D (arrow) 3A8-C12 YFV monoclonal antibody. Secondary antibody conjugated with HRP and reacted with BCIP/NBT substrate.
